# Effect of Targeted Cytokine Inhibition on Progression of Post-Traumatic Osteoarthritis Following Intra-Articular Fracture

**DOI:** 10.3390/ijms241713606

**Published:** 2023-09-02

**Authors:** Michael S. Valerio, Jorge B. Edwards, Connor P. Dolan, Jessica M. Motherwell, Benjamin K. Potter, Christopher L. Dearth, Stephen M. Goldman

**Affiliations:** 1 Research & Surveillance Division, DoD-VA Extremity Trauma and Amputation Center of Excellence, Bethesda, MD 20814, USA; 2 Department of Surgery, Walter Reed National Military Medical Center, Uniformed Services University of the Health Sciences, Bethesda, MD 20814, USA; 3 Department of Orthopaedic Surgery, Walter Reed National Military Medical Center, Bethesda, MD 20889, USA

**Keywords:** articular cartilage, intra-articular fractures, post-traumatic osteoarthritis, synovial fluid, interleukin 1 receptor antagonist, tumor necrosis factor inhibitors, inflammation

## Abstract

Intra-articular fractures (IAF) result in significant and prolonged inflammation, increasing the chances of developing post-traumatic osteoarthritis (PTOA). Interleukin-one beta (IL-1β) and Tumor Necrosis Factor-alpha (TNF-α) are key inflammatory factors shown to be involved in osteochondral degradation following IAF. As such, use of targeted biologics such as Infliximab (INX), a TNF-α inhibitor, and Anakinra (ANR), an interleukin-one (IL-1) receptor antagonist (IL1RA), may protect against PTOA by damping the inflammatory response to IAF and reducing osteochondral degradation. To test this hypothesis, IAFs were induced in the hindlimb knee joints of rats treated with INX at 10 mg/kg/day, ANR at 100 g/kg/day, or saline (vehicle control) by subcutaneous infusion for a period of two weeks and healing was evaluated at 8-weeks post injury. Serum and synovial fluid (SF) were analyzed for soluble factors. In-vivo microcomputed tomography (µCT) scans assessed bone mineral density and bone morphometry measurements. Cationic CA4^+^ agent assessed articular cartilage composition via ex vivo µCT. Scoring according to the Osteoarthritis Research Society International (OARSI) guidelines was performed on stained histologic tibia sections at the 56-day endpoint on a 0–6 scale. Systemically, ANR reduced many pro-inflammatory cytokines and reduced osteochondral degradation markers Cross Linked C-Telopeptide Of Type II (CTXII, *p* < 0.05) and tartrate-resistant acid phosphatase (TRAP, *p* < 0.05). ANR treatment resulted in increased chemokines; macrophage-chemotractant protein-1 (MCP-1), MPC-3, macrophage inhibitory protein 2 (MIP2) with a concomitant decrease in proinflammatory interleukin-17A (IL17A) at 14 days post-injury within the SF. Microcomputed tomography (µCT) at 56 days post-injury revealed ANR Treatment decreased epiphyseal degree of anisotropy (DA) (*p* < 0.05) relative to saline. No differences were found with OARSI scoring but contrast-enhanced µCT revealed a reduction in glycosaminoglycan content with ANR treatment. These findings suggest targeted cytokine inhibition, specifically IL-1 signaling, as a monotherapy has minimal utility for improving IAF healing outcomes but may have utility for promoting a more permissive inflammatory environment that would allow more potent disease modifying osteoarthritis drugs to mitigate the progression of PTOA after IAF.

## 1. Introduction

Intra-articular fractures (IAFs) pose a significant challenge in joint injury treatment as they often result in post-traumatic osteoarthritis (PTOA) [1]. In fact, the likelihood of developing PTOA is over 20 times higher with an IAF than with other, less severe joint injuries [2]. IAFs are typically high energy mechanical injuries and are more prevalent among younger populations, particularly within the military [3]. Consequently, these young patients suffer from chronic joint problems and experience a reduced quality of life [4]. Additionally, the long-term treatment costs for these individuals are exceptionally high for both military and civilian healthcare systems [5].

PTOA is characterized by joint incongruences and chronic inflammation, leading to progressive joint degeneration beyond the initial injury and disrupting the synovial and osteochondral environments [1]. Understanding the role of the immune-inflammatory response in driving PTOA progression is of great interest. Proinflammatory cytokines such as Tumor Necrosis Factor (TNF) and Interleukin IL-1β have been directly implicated in PTOA pathology [6,7,8,9]. Accordingly, there is a strong desire for therapeutic approaches that modulate the soluble factor microenvironment to promote improved long-term outcomes. In response to this need, two biologics have been developed: (1) Infliximab (INX), a TNF-α inhibitor [10], and (2) Anakinra (ANR), an IL-1 receptor antagonist (IL1RA) [11], which may protect against inflammation-induced joint degeneration.

INX is a monoclonal antibody therapy that inhibits TNF-α mediated signaling, showing efficacy in reducing cartilage degradation and bone resorption in osteoarthritis [12] and rheumatoid arthritis [13]. In a preclinical rat model of PTOA, TNF inhibition was found to be chondroprotective by decreasing sulfated glycosaminoglycans (sGAG) release and increasing cartilage lubricin content [14]. However, the effects of INX have been inconsistent, with some studies indicating no improvement in chronic arthritis or metabolic profiles of rheumatoid arthritis patients [15,16]. Further investigation is required to determine the optimal timing and treatment strategy for TNF inhibition, considering patient demographics and the extent of the injury.

ANR, similar to INX, has shown protective effects against rheumatoid arthritis and osteoarthritis [17,18]. While the pathogenesis of rheumatoid arthritis, osteoarthritis, and PTOA differ, the common contributions of the immune-inflammatory response suggests that ANR may have potential for mitigating PTOA associated with IAF. In fact, Furman et al. found that IL-1 inhibition was more effective than TNF inhibition and that local delivery of ANR through intraarticular injections prevented post-traumatic arthritis, while systemic delivery worsened their pathology [19]. They suggested that systemic and long-term administration of IL1RA affected cellular recruitment and altered the healing response. Intraarticular delivery of IL1RA protected against PTOA in their model, but systemic treatment may reduce the risk of synovitis and potential synovial infection following IAF. However, use of intraarticular delivery of drugs has been met with mixed effects due to rapid drug clearance, thus potentially reducing effectiveness [20]. Therefore, we aimed to deliver ANR subcutaneously at a lower dose and only during the inflammatory phase to mitigate the negative effects of systemic administration reported previously.

The purpose of this study was to examine the impact of administering ANR and INX systemically during the inflammatory phase of wound healing on the outcomes of IAF and the progression of PTOA. Our hypothesis was that these biologic therapies would reduce both local and systemic inflammatory responses associated with IAF, ultimately mitigating osteochondral degradation. The findings from this study have the potential to guide clinical practice by determining the potential efficacy of treating IAF injuries with these agents.

## 2. Results

### 2.1. Fracture Characteristics

**Figure 1:**
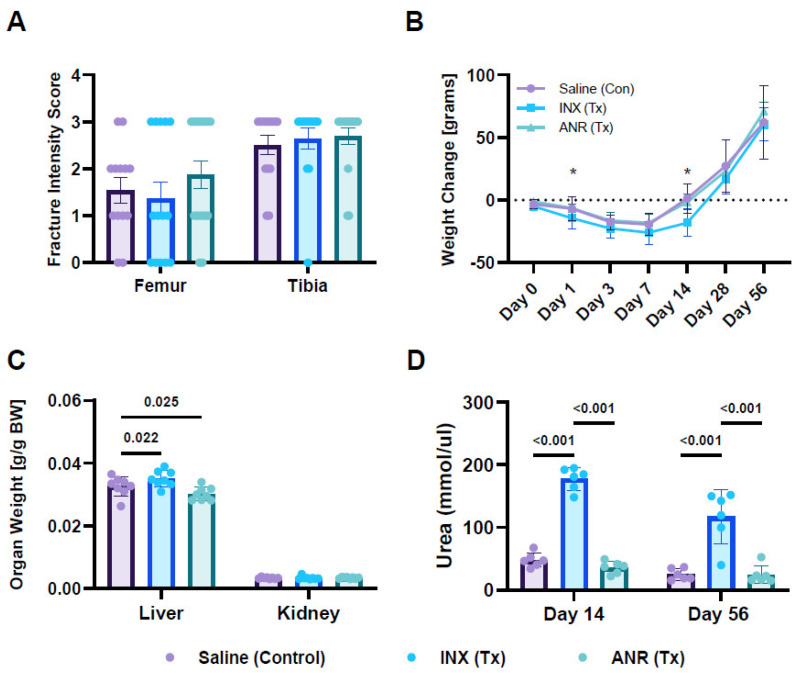
Assessment of Fracture Induction, Body/Organ Weights and Biofluid Changes Following IAF and Treatment with ANR. (**A**) Intensity score of induced IAF. (**B**) Change in body weight over time, normalized to pre-injury starting weight. (**C**) Terminal (Day 56) endpoint organ weight, normalized to endpoint body weight. (**D**) Urea content measured from isolated synovial fluid samples. Treatment groups were saline (Control), INX (Tx) and ANR (Tx). Values are the mean ± SEM of n = 6–8 biologic replicates. *p*-values are listed on each figure section unless otherwise indicated. * *p* < 0.05.

Fractures were predominantly classified as 33C and 41C for femurs and tibias, respectively, with many animals exhibiting injuries which affected both long bones of the knee joint.There were no differences noted in the type or severity of fracture between groups (Figure 1A).

### 2.2. Systemic Changes Following Treatment with INX and ANR

Changes in body weights (normalized to pre-injury weights) revealed that all animals lost weight following injury through seven days, followed by recovery to baseline weights at D14 and growth through Day 56 (Figure 1B). Comparing weight change per timepoint revealed a reduction in body weights for the INX group at Day 1 (*p* < 0.05) and at D14 (*p* < 0.001) compared to controls. However, all differences were lost after Day 14. ANR did not show any changes in body weight compared to control. In addition to changes in body weight, liver and kidney weights were also measured in INX and ANR treated animals compared to control (Figure 1C). Analysis of organ weights revealed an increase in liver weight with INX treatment (*p* < 0.05) while ANR reduced liver weights (*p* < 0.05). No changes in kidney weight were observed between groups. Moreover, SF samples were analyzed for urea content. Preliminary results from INX treated animals revealed an increase in urea content (~4-fold, *p* < 0.001) in the SF compared to saline and ANR treatment (Figure 1D). As a result of the confounding systemic effects of INX, only data from Saline or ANR treated animals were analyzed downstream.

### 2.3. Effect of IL-1 Inhibition on Systemic Soluble Factor Expression Following IAF

**Figure 2:**
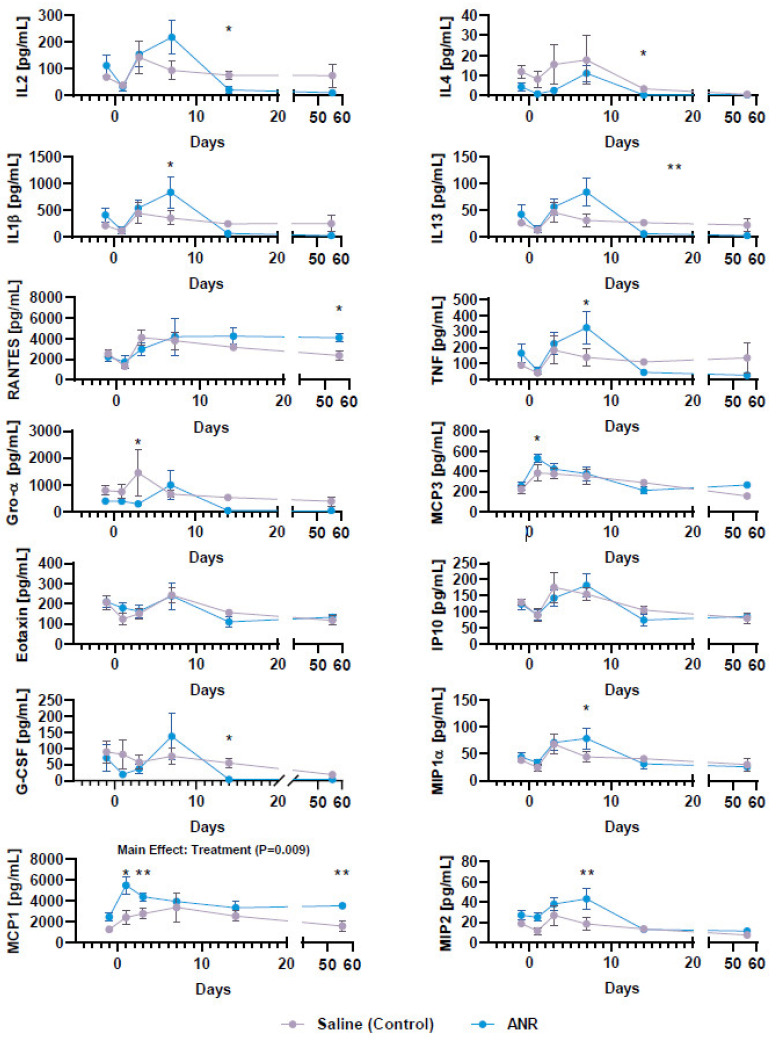
Systemic Cytokine and Chemokine Expression Following IAF and IL-1 Inhibition over Time. Cytokine and chemokine expression from serum collected from saline (con) and ANR (Tx) treated animals on days -1, 1, 3, 5-, 7-, 14- and 56-days post-injury. Cytokines/Chemokines include IL-1β, IL-2, IL-4, IL-13, TNFα, G-CSF, Eotaxin, Gro-α, IP-10, RANTES, MCP-1, MCP-3, MIP1α, and MIP2. Values were corrected for dilution factor and a thresholding was applied based on expression relative to lowest quartile of detection limits. Values are the mean ± SEM of n = 6 biologic replicates. * *p* < 0.05, ** *p* < 0.01.

The expression levels of various cytokines and chemokines were analyzed to investigate the systemic inflammatory response (Figure 2). ANR treatment was found to decrease the expression of IL-1β (~3.5-fold, *p* < 0.05), IL-2 (~4-fold, *p* < 0.05), IL-13 (>4-fold, *p* < 0.01), CXCL1 (~5-fold, *p* < 0.05), and G-CSF (>15-fold, *p* < 0.05), but not IL-4, at Day 14 post-injury. Expression of TNFα was found to be increased by ANR treatment (>2-fold, *p* < 0.05). Likewise, ANR treatment was also found to influence the expression levels of MCP-1 (CCL2) at all time points, MCP-3 (CCL7) at Day 3 post-injury, MIP1a (~2-fold, *p* < 0.05) and MIP2 (over 2-fold, *p* < 0.05) at Day 7 post-injury, and RANTES (CCL5) at Day 56 post-injury, all relative to saline controls.

### 2.4. Effect of IL-1 Inhibition on Systemic Expression of Osteochondral Markers Following IAF

**Figure 3:**
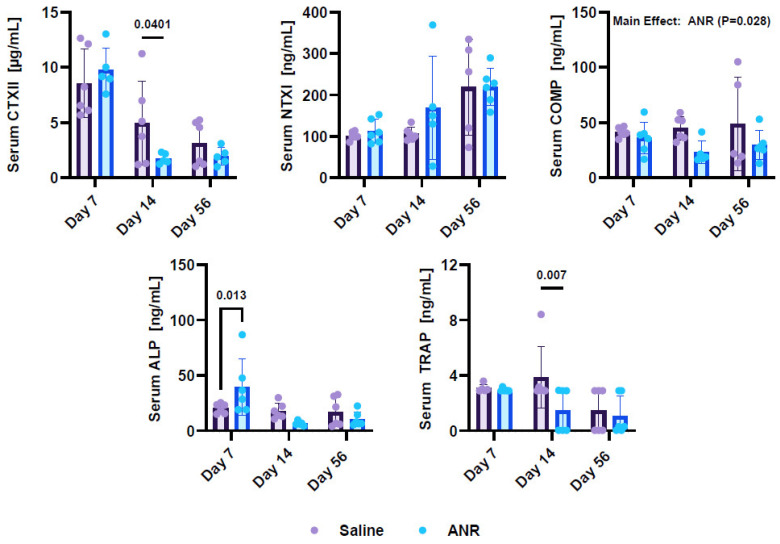
Systemic Osteochondral Marker Expression in Response to IAF and IL-1 Inhibition over Time. Osteochondral marker expression from serum collected from saline (Con) and ANR (Tx) treated animals on days 7, 14 and 56 post-injury. Osteochondral markers include CTXII, NTX1, COMP, ALP and TRAP. Values were corrected for dilution factor and a thresholding was applied based on expression relative to lowest quartile of detection limits. Values are the mean ± SEM of n = 6 biologic replicates. *p*-values are listed on each figure section unless otherwise indicated.

Osteochondral turnover markers were measured in serum throughout the wound healing timeline (Figure 3). Osteoblast activity, as measured by ALP expression, was shown to be increased (*p* = 0.013) at Day 7 post-injury, compared to the saline control group, but there was no difference between groups at later timepoints (Day 14 and Day 56). Osteoclast activity, as measured by TRAP expression, was shown to be reduced (*p* = 0.007) at day 14 post-injury in the ANR-treated group relative to the saline control group. Serum COMP, a measure of cartilage breakdown, was reduced with ANR treatment across all study endpoints (Main Effect, *p* = 0.028). NTX1, a measure of bone breakdown, was not differentially expressed between groups over time, but was increased as a function of time (*p* = 0.002). Serum CTXII was reduced with ANR at day 14 post-injury (*p* = 0.040).

### 2.5. Effect of IL-1 Inhibition on Local Synovial Fluid Expression of Inflammatory and Osteochondral Markers Following IAF

**Figure 4:**
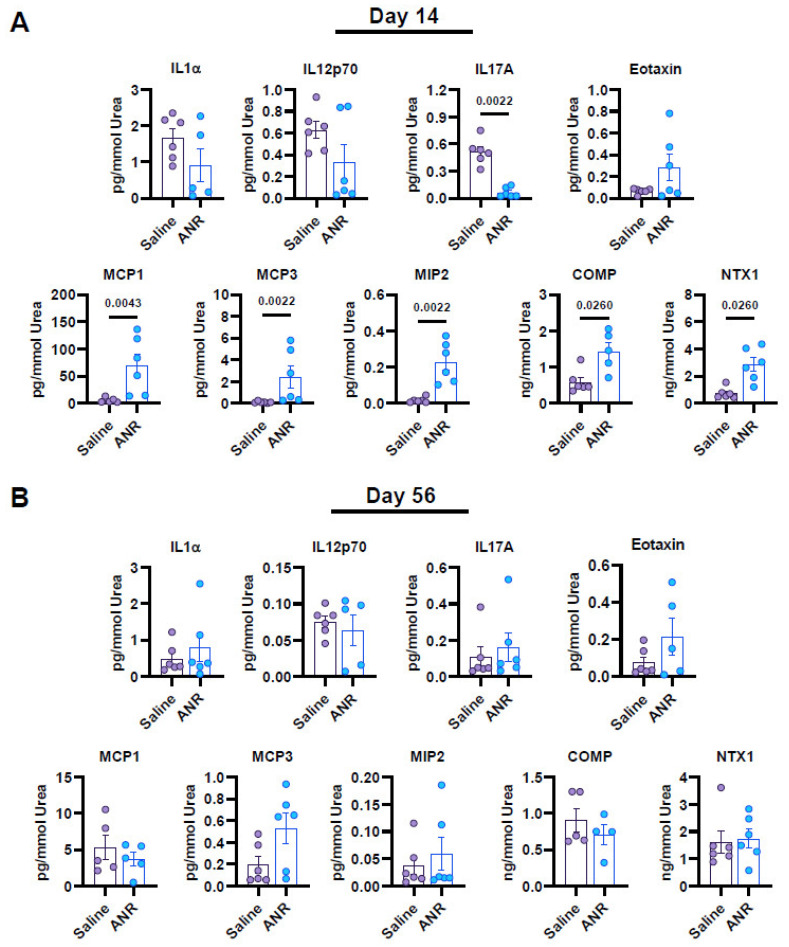
Local Inflammatory and Osteochondral Marker Expression from Synovial Fluid Following IAF and IL-1 Inhibition at Early (Day 14) and Late (Day 56) Terminal Endpoints. Cytokine/Chemokine and osteochondral maker expression from synovial fluid samples collected from saline and ANR groups at Day 14 (**A**) and Day 56 (**B**) and normalized to total urea. Markers at Day 14 include IL-1α, IL-1β, IL-10, IL-12p70, IL-17A, Eotaxin, MCP-1, MCP-3, MIP2, COMP and NTX1. Markers at Day 56 include IL1α, IL-12p70, IL-17A, Eotaxin, MCP-1, MCP-3, MIP2, COMP and NTX1. Values were corrected for dilution factor and a thresholding was applied based on expression relative to lowest quartile of detection limits. Values are the mean ± SEM of n = 6 biologic replicates. *p*-values are listed on each figure section unless otherwise indicated.

ANR treatment was found to reduce the expression of IL-17A (*p* < 0.01) at Day 14, compared to the saline control group (Figure 4). It was also found to increase the expression of chemokines MCP-1 (*p* < 0.01), MPC-3 (*p* < 0.01) and MIP2 (*p* < 0.01) as well as osteochondral breakdown markers COMP (*p* < 0.5), and NTX1 (*p* < 0.05). Data analyzed from Day 56 samples revealed no differences between saline and ANR treatment.

### 2.6. Effect of IL-1 Inhibition on Bone Mineral Density

**Figure 5:**
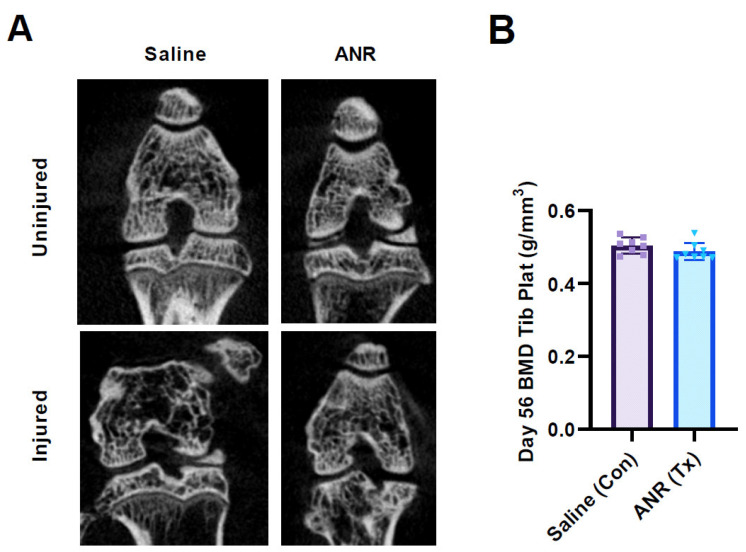
Representative µCT scans and Bone Mineral Density (BMD) at Terminal (Day 56) Endpoint. (**A**) Representative reconstructed in vivo µCT scans cut in the coronal plane of the entire articular joint from uninjured and injured Saline, uninjured and injured ANR treated animals. (**B**) Bone mineral density measured at the segmented tibial epiphysis (Plateau) from saline (Con) and ANR (Tx) treated animals. Dotted line represents mean BMD values of contralateral limbs. Values are the mean ± SEM of n = 6 biologic replicates. *p*-values are listed on each figure section unless otherwise indicated.

Bone mineral density in the tibial epiphysis was analyzed from μ-CT images at eight weeks post-injury to assess differences in bone healing (Figure 5). Coronal sections that highlight the qualitative differences in the form and structure of injured and uninjured bones are evident (Figure 5A). Moreover, the images highlight the differences between treatment groups in the uninjured limbs. Figure 5B shows the differences in BMD between the contralateral (uninjured) and injured treatment groups. There were no differences in BMD within the subchondral bone between groups. Interestingly, there were no differences noted between the Saline (control) injury group and the contralateral limb with respect to BMD.

### 2.7. Effect of IL-1 Inhibition on Bone Morphometry

µ-CT scans were analyzed for changes in trabecular bone morphometric measurements (BMM) amongst the experimental groups (Table 1). Relative to uninvolved contralateral limbs, our saline controls group exhibited alterations (*p* ≤ 0.05) in a majority of the metric analyzed. Notably, bone volume (BV), bone volume fraction (BV/TV), bone surface area (BS), trabecular number (TB.N), and connectivity (Conn) were all reduced in the saline controls relative to the contralateral limbs while trabecular thickness (Tb.Th) and degree of anisotropy (DA) were increased in the saline controls. ANR treatment resulted in increases in BV, tissue surface (TS) and Tb.Th with a concomitant decrease in DA.

### 2.8. Effect of IL-1 Inhibition on Histopathological Presentation of Joint Tissues

**Figure 6:**
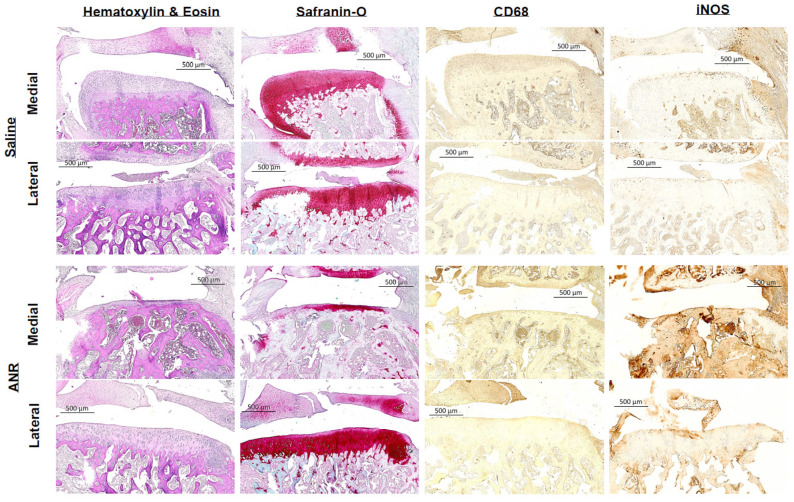
Histological/Immunohistochemical Sections Following IAF and IL-1 Inhibition at Early Terminal (Day 14) Endpoint. Left Tibias harvested at Day 14 post-injury. Top half of panel represents images from medial and lateral stained tissues harvested from Saline treated animals. Bottom half of panel represents medial and lateral aspects of stained tissues harvested from ANR treated animals. Panels were stained for cellularity (H&E), sGAG (Saf O), pan macrophage (CD68) and M1 expression (iNOS). Images are representative of n = 6 biologic replicates.

**Figure 7:**
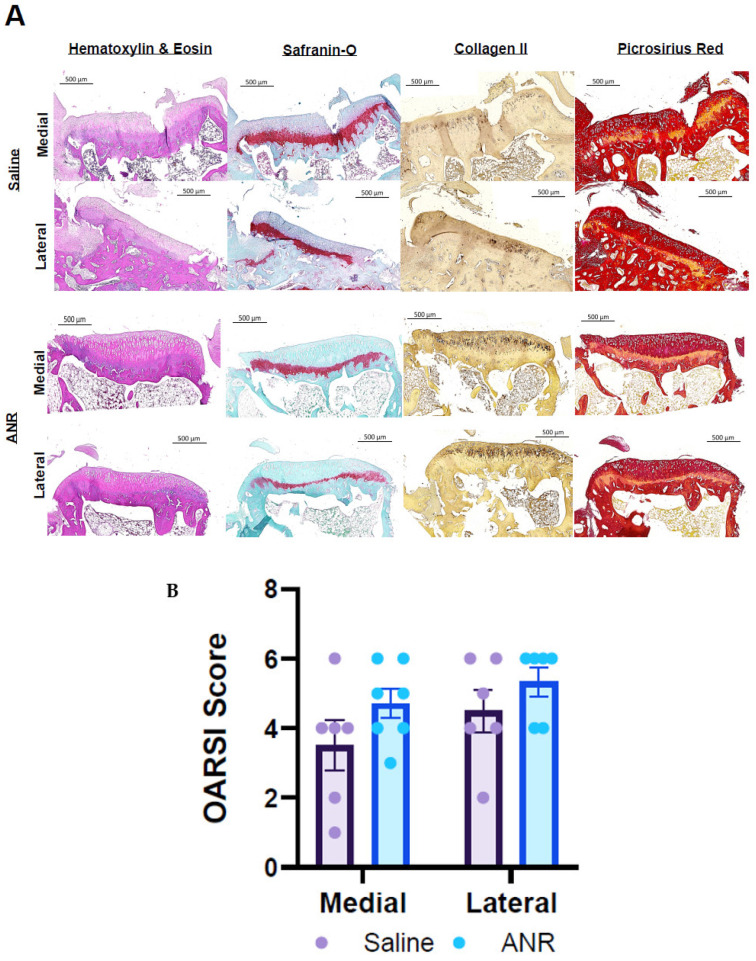
Histological/Immunohistochemical Sections and Clinical Score Following IAF and Il-1 Inhibition at Late Terminal (Day 56) Endpoint. (**A**) Left Tibias harvested at Day 56 post-injury. Top half of panel represents images from medial and lateral stained tissues harvested from Saline treated animals. Bottom half of panel represents medial and lateral aspects of stained tissues harvested from ANR treated animals. Panels were stained for cellularity (H&E), sGAG (Saf-O), Collagen 2 (Col2) and fibrosis (Picrosirius Red). Images are representative of n = 6 biologic replicates. (**B**) Composite OARSI scores of joints based on H&E and Safranin O staining. Values are the mean ± SEM of n = 6 biologic replicates. *p*-values are listed on each figure section unless otherwise indicated.

Histological analysis of samples at 14 days post-injury (Figure 6) revealed less inflammatory infiltration via H&E staining and more robust sGAG content via Safranin-O staining in the ANR treatment group relative to the control group. Staining for CD68 and iNOS suggests a qualitative reduction in macrophage abundance and pro-inflammatory activity with the ANR treatment group relative to the saline controls. Analyses at 56 days post-injury (Figure 7) revealed greater calcified cartilage in the medial and lateral aspects of the articular cartilage in the saline treated group compared to the ANR control group. Safranin O staining revealed an overall reduction in sGAG in both Saline and ANR treated groups. Staining for Type II collagen did not reveal any qualitative differences between groups. OARSI scoring of the 56-day samples revealed no differences between the Saline and ANR treatment groups (Figure 7).

### 2.9. Effect of IL-1 Inhibition on IAF Cartilage Content Measured Ex Vivo by CECT

**Figure 8:**
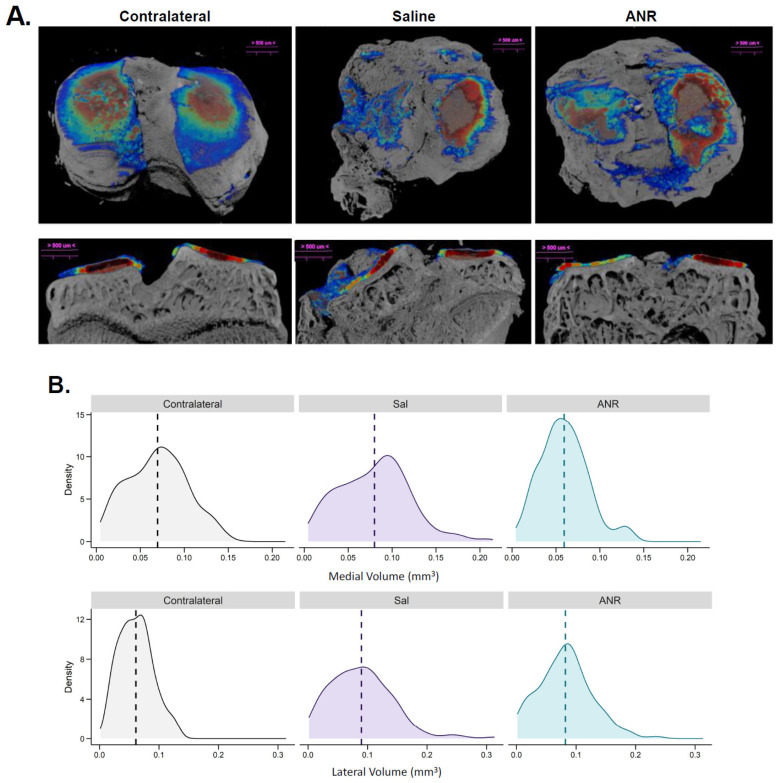
Contrast Enhanced Assessment of Sulfated Glycosaminoglycans at Terminal (Day 56) Endpoint. (**A**) Left tibial µCT scans with contrast enhanced cationic CA4+ density map overlay from Contralateral, Saline and ANR treated animals. Top panel is representative scans in transaxial orientation. Bottom panel is representative coronal orientation of the same scan. Thin sections are highlighted in blue and increasing in thickness to deep red. Images are representative of n = 6 biologic replicates. (**B**) Quantitation of CA4^+^ volume and density distribution from each group.

Contrast-enhanced µCT scans (Figure 8A) were used to map the density distribution of sGAGs within the articular surface of tibias at 56 days post-injury. The results show that IAF induced changes to the sGAG density and distribution in both groups, compared to the contralateral limbs. Binning density distribution as a function of volume (Figure 8B) reveals that IAF induces changes in sGAG density distribution, and ANR treatment does not alter this basal outcome. The medial density vs volume plot shows that saline treatment increased the volume distribution but reduced the overall density of the cartilage, whereas with ANR treatment, there was a slight shift to the left indicating less volume overall but with a large increase in density at all volume measures. The lateral (bottom) panel shows that the ANR treatment increased overall density relative to saline and was like the contralateral measurements.

## 3. Discussion

This study aimed to examine the impact of two drugs, INX and ANR, on the healing process of IAF and the development of PTOA. Previous research has shown varying degrees of success in preserving and restoring joint health through targeting the TNF and IL1 pathways [20,21]. Inconsistencies in the outcomes of these treatments might be attributed to differences in the timing and frequency of administration. During the normal wound healing process, the inflammatory phase transitions into the anabolic phase, enabling cells to regenerate damaged tissue [22]. This transition is proportional to the extent of tissue damage and inflammation caused by the injury. However, in the case of IAF, the prolonged and overactive inflammatory response hampers the normal wound healing process by impeding the functioning of mesenchymal stem cells (MSCs) leading to suboptimal repair outcomes characterized by fibrous tissue deposition [22,23].

Based on the heightened inflammatory phase observed following IAF, the study proposed that administering treatments systemically only during the initial two weeks after injury (referred to as the inflammatory phase) would yield overall positive effects on osteochondral healing. The goal was to suppress the excessive immune response triggered by IAF, allowing cells to regenerate damaged tissue, and thereby protecting against PTOA.

During the initial phase of the study, the data revealed weight loss and concurrent increases in liver weight in subjects receiving INX treatment. These results were consistent with previous research indicating that INX treatment could lead to liver dysfunction [24]. Additionally, it was found that INX had minimal impact on both systemic inflammation and the healing metrics of IAF. Unexpectedly, INX treatment resulted in a notable increase in serum urea levels, suggesting potential metabolic dysfunction. This finding aligned with clinical reports indicating that INX could cause liver dysfunction, heightened knee pain, and elevated urea levels [16,25]. Consequently, INX was excluded as a viable treatment option for IAF injuries moving forward due to this confounding variable.

After excluding INX as a potential treatment option, our attention shifted towards investigating the effects of systemic administration of ANR during the inflammatory phase of wound healing. For this study, we chose to deliver the lower dose (100 µg/kg/day), continually over 14 days to target the inflammatory phase of wound healing and only slightly suppress the immune response as a result of IAF. Inflammation during wound healing is necessary and it has been shown that over suppression of the inflammatory response reduces healing [26]. Therefore, the dose we chose represents the lower level effective dose scaled down from the suggested human dose (100 mg/day), which was shown to be effective in reducing RA [17]. Notably, we observed that our treatment with ANR resulted in significant reductions in the expression of IL-1β, IL-2, IL-13, CXCL1, and G-CSF in the serum after 14 days of injury. However, ANR treatment led to an increase in TNFα expression. Furthermore, ANR treatment influenced the levels of MCP-1, MCP-3, MIP1a, MIP2, and RANTES in the serum at various time points following the injury, when compared to the control group treated with saline.

G-CSF is a chemotactic cytokine that plays a crucial role in guiding neutrophils to the wound site after an injury, contributing to the initial phase of wound healing. These findings support our hypothesis that sustained early administration of ANR will attenuate the immune response by reducing the number of innate immune cells, thus facilitating tissue regeneration. Furthermore, qualitative analysis of images taken on Day 14 revealed a decrease in infiltrating CD68 and iNOS positive cells in the ANR-treated group compared to the control group. These results further support our hypothesis.

Consistent with these findings, ANR also created a chemotactic environment that facilitated fracture healing. Specifically, ANR treatment increased the expression of MCP-1, MCP-3, and MIP2 on Day 14 after the injury. MCP-1 (CCL2) is known to regulate fracture healing and bone remodeling by promoting the expansion of MSCs involved in osteochondral healing. Ishiwaha demonstrated that MCP-1 is crucial in the early stages of fracture healing [27]. MCP-3 (CCL7) has been identified as a factor that attracts MSCs and has been shown to increase following trauma [28]. Lastly, MIP2 (CXCL2) has been found to be up-regulated in the synovial fluid on Day 14 after the injury. MIP2 (CXCL2) acts as a potent chemoattractant for neutrophils and is known to be up-regulated following an injury. It may also play a role in angiogenesis during wound healing and promote macrophage switching from the M1 to the M2 phenotype [29].

Although our study demonstrated that ANR treatment reduced the levels of another neutrophil chemoattractant (G-CSF), these findings are not contradictory. Previous reports indicate that G-CSF is involved in early neutrophil recruitment and actually inhibits CXCL2/CXCR2-induced neutrophil recruitment [30]. Therefore, suppressing G-CSF and enhancing CXCL2 activity may help alleviate the inflammatory burden and promote healing. Consequently, further investigation is warranted to explore the impact of ANR on CXCL2 expression following IAF.

In addition to its effects on the inflammatory and MSC environment, ANR treatment had a significant impact on reducing systemic IL-2 expression following IAF, which is consistent with a previous study by Pham et al. [7]. IL-2 plays a role in regulating T regulatory cells and inducing an adaptive immune response associated with various inflammatory diseases. Interestingly, Yokoyama found that treatment with intravenous injections of anti-IL-2 antibodies in a collagen-induced arthritis (CIA) model led to a reduction in synovial destruction by modulating T cell function [31,32]. This supports the notion that T cell function plays a crucial role in synovial destruction in conditions like rheumatoid arthritis (RA) and CIA [33]. Additionally, Faust et al. demonstrated that administration of IL-17A neutralizing antibodies improved the development of PTOA in an ACL transection model [32]. Our findings align with these studies and demonstrate that IL-2 expression, a marker of T cell function, was elevated in synovial fluid samples treated with saline following IAF, but significantly reduced with ANR treatment. These results further support the earlier observation of a decrease in systemic IL-2 expression with ANR treatment. Our study shares similarities with the work conducted by Olson et al., who examined the impact of systemic versus local administration of ANR as a treatment to prevent the development of PTOA following IAF [19]. In their study, they discovered that continuous systemic administration of ANR via osmotic pumps for 28 days, using clinically relevant doses, had negative effects on IAF wound healing. In fact, the authors concluded that “prolonged systemic delivery of IL-1Ra does not provide therapeutic benefits in this model” [34]. In contrast to Olson et al.’s approach, our study focused on administering ANR during the initial two weeks after injury. We found that ANR treatment effectively reduced the systemic expression of its primary target, the pro-inflammatory cytokine IL-1β, 14 days after the injury.

Regarding osteochondral healing, previous research by Kim et al. has demonstrated that IL-1 plays a crucial role in activating osteoclasts [35]. Conversely, blocking IL-1 has been shown to reduce joint destruction caused by osteoclasts [36]. Our data support these findings by indicating that ANR treatment reduced cartilage breakdown, as evidenced by lower levels of the cartilage breakdown markers CTXII at day 14 after the injury and COMP throughout all endpoints. Moreover, ANR treatment increased the serum marker of osteoblast activity, ALP, at day 7 post-injury, while decreasing the serum marker of osteoclast activity, TRAP, at day 14 post-injury. Collectively, these results suggest that ANR had an overall positive effect on healing intra-articular fractures (IAF) by protecting against cartilage breakdown and providing some evidence of bone healing.

While our study provides evidence supporting the beneficial role of ANR in healing IAF, our data revealed some intriguing findings. Specifically, we observed an increase in TNF-alpha levels with ANR treatment at Day 7 after the injury, but this increase was not observed at any subsequent time points. Additionally, we found up-regulation of COMP, a marker of bone loss, and NTX1, a marker of chondrogenic degradation, in the serum at Day 14 after the injury, but not at the terminal endpoint (Day 56).

These findings suggest that ANR may have a complex impact on osteochondral healing, with different factors influencing its expression at different stages of the healing process. One possible explanation for the differences in early and late expression of these markers could be the extent of the wound. ANR is believed to affect the healing of both cartilage and subchondral bone in intra-articular fractures. Our measurements of BMD revealed that ANR treatment significantly reduced BMD in the tibial shaft, while no differences were observed in the plateau region. Additionally, our measurements of BMM showed no significant differences in the trabecular structure of the epiphysis between the saline and ANR treatment groups. Furthermore, the OARSI scores at Day 56 showed no major differences between the groups. In contrast, our CECT data indicated an enhancement of sGAGs in the ANR-treated group compared to the controls.

Therefore, the variable effects of ANR on osteochondral healing may be attributed to the broad nature of the injury model and the specific mechanisms through which ANR operates. Further research is needed to gain a comprehensive understanding of the complex effects of ANR on osteochondral healing. Alternatively, the intensity of fractures induced by our model does pose a limitation to this study as finer effects from ANR may have been lost due to the nature of injuries sustained by impact-induced IAF. This can be addressed in future studies by evaluating the effects of ANR in joint models of varying levels of injury severity.

These findings indicate that targeted inhibition of cytokines, particularly IL-1 signaling through ANR, may effectively reduce the inflammatory response triggered by IAF. However, our results suggest that the extent of damage in our IAF model might have compromised some of the beneficial effects of ANR. Nonetheless, our findings align with previous studies utilizing different models, which have demonstrated varied effects of inhibiting IL-1 signaling on the health of articular joints. Based on our results, future studies will focus on optimizing the administration of pro-regenerative small molecules in combination with ANR. This approach holds promise as a potential strategy for enhancing the healing outcomes of IAF.

## 4. Materials and Methods

### 4.1. Preparation of Interventions

Osmotic pumps (Alzet, DURECT, Cupertino, CA, USA) were used to deliver INX (Remicade, Janssen Biotech, Beerse, Belgium) and ANR (Kineret, Sobi Biopharmaceuticals, Stockholm, Sweden). Working concentrations of 10 mg/kg/day and 100 µg/kg/day for INX and ANR, respectively, were prepared in 0.9% physiologic saline, and pumps were filled and primed overnight at 37 °C prior to implantation.

### 4.2. Intra-Articular Fracture Model and Drug Delivery

**Figure 9:**
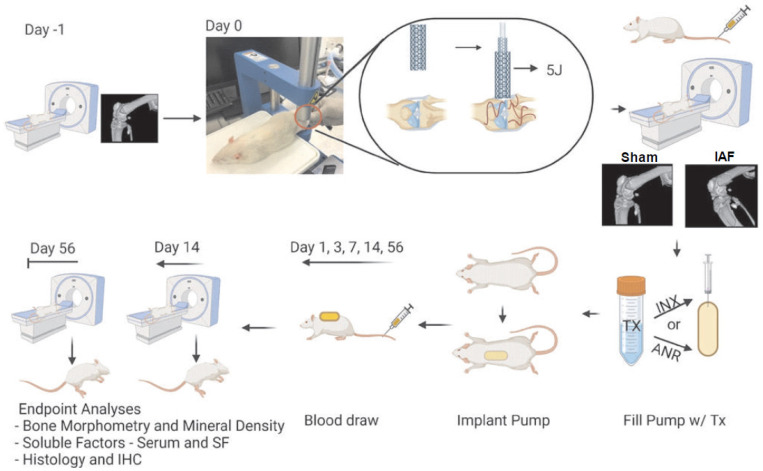
Induction of Intraarticular Fracture, Treatment Procedures and Data Collection Schematic. Workflow indicates µCT scans will be performed on Day-1 (Pre-injury), immediately following IAF and at terminal endpoints (Days 14 and 56) as indicated on the workflow diagram. On Day 0, IAF is induced by a 5 J impact energy on the lateral aspect of an isolated hind limb. Pumps filled with Saline (Con), INX (Tx) or ANR (Tx) are primed and implanted subcutaneously for 14 days following injury with blood draws at 1, 3, 5-, 7-, 14- and 56-days post injury.

This study received approval from the Institutional Animal Care and Use Committee at the Uniformed Services University of the Health Sciences. Male Lewis rats (n = 8/group, 300–350 g) were obtained from Charles River Labs (Charles River, NJ, USA) and were housed under standard animal husbandry protocols with ad libitum access to food and water. To monitor the impact of injury and drug intervention, the body weights (BW) of the rats were measured throughout the study. The rats were anesthetized (2–4% isoflurane) and given a single injection of buprenorphine SR at a concentration of 1 mg/kg body weight for pain management. The IAF was induced by delivering a 5 J blunt impact to the lateral aspect of the left limb, and its induction was verified through micro-computed tomography (µCT) (Figure 9). After injury induction, osmotic pumps were implanted in the subcutaneous space along the rat dorsum. The pumps were block-randomized into different experimental groups to deliver Saline (vehicle control), INX, or ANR continuously for 14 days and were aseptically explanted after the 14-day period.

### 4.3. Tissue, Blood, and Synovial Fluid Harvest

Whole blood was collected from the tail artery of rats prior to injury and at various timepoints (1, 3-, 7-, 14-, and 56-days post-injury) during the course of experimentation. After collection, serum was obtained from whole blood by centrifugation at 1000 RPM for 10 min at room temperature, and snap-frozen in liquid nitrogen for later analysis. Animals were euthanized at days 14 and 56 by intracardiac injection of 10% Euthasol while under general anesthesia. The patellar tendon was transected and reflected away to provide access to the synovium. Synovial fluid (SF) was collected by making a small incision in the synovium and absorbing the fluid with pieces of Whatman paper [37]. SF samples were then snap-frozen and the hindlimbs were disarticulated and placed in 10% formalin for histological analysis.

### 4.4. Micro-Computed Tomography

µCT scans (SkyScan 1278, Bruker Corporation, Billerica, MA, USA) were performed to assess the severity of the IAF and to evaluate the healing process at 2- and 8-weeks post-injury. Scans were conducted with a 50 µm resolution using a 1 mm Al filter, 65 kV, 770 µA, 360° rotation, 56 ms exposure, and 0.3° rotation steps. The scans were 10 min long and yielded 400 milligreyes total per scan. The reconstructed images were truncated to isolate only the femur and tibia from each scan, and whole reconstructed 3D images were rendered for visualization and qualitative assessment of the injured and contralateral limbs. Post-reconstruction alignment was achieved using the SkyScan DataViewer (Version 1.5.2.4, Bruker Corporation, Billerica, MA, USA) for qualitative image processing. 3D images were rendered from the reconstructed scans, and a threshold from 6300 to 63,000 was applied to the grey values. The limbs were aligned at 90° angles, and regions of interest (ROI) were obtained for the whole femurs, and tibia/fibula from both the injured and contralateral legs. Volumes of interest were further segmented from the femur and tibia to isolate the epiphyseal and metaphyseal spaces for bone morphometry and bone mineral density measurements. To isolate the femoral and tibial epiphyses, transaxial sections were used to determine landmarks of the distal femur and proximal tibia. These landmarks were marked as slice 1, and then moving proximally 30-slices (150 µm) or from the proximal tibia, and 10 slices (50 µm) distally were used to isolate the epiphyseal sections.

### 4.5. Fracture Assessment

Three-dimensional reconstructions of µCT scans were used to assess IAF severity using the 2018 AOOTA JOT Fracture Classification and Dislocation Compendium [38]. A series of images representing a 360° view of the injured and contralateral limbs were generated and blinded for classification by seven independent raters. Images were scored based on the location of the fracture and level of intensity. Frequency of fractures were noted for both the femur and tibia from each sample and tallied (Figure S1).

### 4.6. Synovial Fluid Protein Extraction

To prepare the SF samples for immunoassays, the frozen samples were thawed and reconstituted in 300 µL of Luminex-compatible cell lysis buffer (ThermoFisher, Waltham, MA, USA). The reconstitution was completed overnight at 4 °C with gentle agitation to desorb the SF from the Whatman paper. Subsequently, the samples were transferred to tissue homogenization tubes (MP Biosciences, Santa Ana, CA, USA) with a ceramic bead and homogenized using a tissue homogenizer. The homogenate was then filtered to remove any remnants of the Whatman paper, and aliquoted for downstream use.

### 4.7. Protein Analysis

To analyze cytokine and chemokine levels in serum and SF samples, a cytokine and chemokine multiplex immunoassay (ProcartaPlex Rat 22 Cytokine/Chemokine Array, Invitrogen, Waltham, MA, USA) was performed according to the manufacturer’s protocol using a BioRad200 Multiplex Plate Reader and Bioplex Manager (Version 6.1, Biorad, Hercules, CA, USA). Samples that did not reach the threshold of detection were counted as one-half of the lowest quartile of detection (LQQ). If more than 50% (or 3 out of 6) of the samples required the one-half LQQ, the entire analyte was excluded from further analysis. For endpoint (Day 14 and 56) serum and SF samples, osteochondral biomarkers COMP, NTX1, ALP, and TRAP using traditional ELISAs (MyBioSource, Inc., San Diego, CA, USA) were analyzed per the manufacturer’s specifications. SF samples were also assayed for urea content (ELISA, mybiosource.com) for normalization purposes to account for variable collection volumes, in accordance with prior reports [39].

### 4.8. Contrasts Enhanced µCT and Analysis

A contrast-enhanced µCT (CECT) method was used to identify disparities in sGAG distribution in the articular surface of the affected hindlimbs. Tibial heads were submerged in 1 mL of a cationic iodized dye, CA4^+^ (12 mg/mL in saline), for 24 h at 4 °C to equilibrate within the tissues. After incubation, tibia heads were blotted dry and scanned on a Bruker 1172 SkyScan µCT with a 0.5 mm Al filter, using 70 kV and 112 uA with an integration time of 300 ms and a voxel size of 6 μm. Scans were reconstructed using a smoothing value of 3, ring artifact reduction of 6, and beam hardening of 20%. To ensure uniformity of angles and rotation and to save a rough volume of interest in a coronal orientation, we oriented the images using DataViewer (Version 1.5.2.4, Bruker Corporation, Billerica, MA, USA). We then analyzed resliced coronal sections using CTAn software (Version 1.15.4.0, Bruker Corporation, Billerica, MA, USA) by selecting the top of the tibial head and then 325 slices down to the growth plate or adjusted accordingly to fracture-induced anatomical changes. To remove trabecular structures and loose soft tissue, we further contoured selected ROIs. We then applied a Gaussian Blur with a radius of 3, filtered with a minimum bandpass filter of 55–110, and eroded the samples in the 2D aspect at a radius of 2.

### 4.9. Histological and Immunohistochemical Analysis

Formalin fixed hindlimbs were rinsed and decalcified with Cal-X (ThermoFisher) solution for a period of 14 days. Once decalcified, the samples were embedded in paraffin blocks for sectioning. Coronal sections measuring five microns were cut and stained with Hematoxylin & Eosin (H&E) and Safranin O for qualitative analysis and OARSI scoring. Picrosirius red staining was employed to visualize collagen intensity as previously described [40]. Immunohistochemistry (IHC) was employed to measure macrophage infiltration and activity at 14 days post-injury via CD68 (ThermoFisher) and iNOS (Abcam, Cambridge, UK), respectively. For Day 56 samples, IHC was performed to analyze Type II collagen (Abcam).

### 4.10. Statistical Analysis

Study outcomes were compared by analysis of variance (ANOVA) with Holm-Šídák post-hoc tests and are reported as the mean ± SEM with a significance level set at α = 0.05 (Graphpad Prism 9.4).

## Figures and Tables

**Table 1:** Effect of Interleukin 1 Inhibition on Tibial Trabecular Bone Morphometric Measurements from in vivo µCT.

Measurement	ID	Unit	Contralateral Mean ± SEM	Saline Mean ± SEM	ANR Mean ± SEM	*p*-Value		
						CTRL vs. Saline	CTRL vs. ARN	Saline vs. ARN
Tissue volume	TV	mm^3^	21.48 ± 0.72	17.00 ± 1.27	22.22 ± 2.44	0.060	0.696	0.058
Bone volume	BV	mm^3^	5.54 ± 0.16	3.66 ± 0.47	4.57 ± 0.46	<0.001	0.036	0.083
Bone volume fraction	BV/TV	-	25.84 ± 0.24	21.57 ± 2.29	20.82 ± 1.20	0.012	0.004	0.686
Tissue surface	TS	mm^2^	91.15 ± 2.81	73.40 ± 5.08	94.04 ± 9.64	0.024	0.700	0.023
Bone surface	BS	mm^2^	103.69 ± 1.77	65.41 ± 7.56	78.44 ± 7.38	<0.001	<0.001	0.104
Intersection surface	i.S.	mm^2^	13.06 ± 0.56	9.08 ± 1.24	11.34 ± 1.28	0.004	0.189	0.138
Specific bone surface	BS/BV	1/mm	18.80 ± 0.20	18.09 ± 0.34	17.31 ± 0.31	0.065	<0.001	0.075
Bone surface density	BS/TV	1/mm	4.86 ± 0.08	3.88 ± 0.39	3.59 ± 0.18	0.001	<0.001	0.376
Trabecular pattern factor	Tb.Pf	1/mm	−3.41 ± 0.17	0.94 ± 0.93	1.95 ± 0.44	<0.001	<0.001	0.193
Structure model index	SMI	-	0.89 ± 0.02	1.39 ± 0.14	1.56 ± 0.06	<0.001	<0.001	0.149
Trabecular thickness	Tb.Th	µm	190.51 ± 1.62	213.19 ± 4.76	223.40 ± 3.56	<0.001	<0.001	0.040
Trabecular number	Tb.N	1/mm	1.36 ± 0.02	1.02 ± 0.12	0.93 ± 0.05	<0.001	<0.001	0.337
Trabecular separation	Tb.Sp	µm	439.24 ± 4.07	457.57 ± 13.97	477.60 ± 8.08	0.105	0.002	0.125
Degree of anisotropy	DA	-	1.79 ± 0.03	2.43 ± 0.12	1.99 ± 0.07	<0.001	0.036	<0.001
Fractal dimension	FD	-	4.02 ± 0.03	3.60 ± 0.15	3.57 ± 0.07	<0.001	<0.001	0.803
Number of objects	Obj.N	-	2.44 ± 0.26	5.88 ± 2.26	9.00 ± 0.98	0.031	<0.001	0.085
Volume of open pore space	Po.V(op)	mm^3^	15.94 ± 0.57	13.34 ± 1.10	17.65 ± 2.10	0.112	0.292	0.026
Open porosity (percent)	Po(op)	%	74.16 ± 0.24	78.43 ± 2.29	79.18 ± 1.20	0.012	0.004	0.686
Euler number	Eu.N	-	−279.60 ± 6.50	−121.00 ± 20.06	−110.00 ± 14.70	<0.001	<0.001	0.560
Connectivity	Conn	-	282.30 ± 6.39	126.90 ± 18.38	119.30 ± 14.68	<0.001	<0.001	0.687

## Data Availability

All data supporting the findings of this study are available within the paper and its Supplementary Information.

## Supplementary Materials

The following supporting information can be downloaded at: https://www.mdpi.com/article/10.3390/ijms241713606/s1.
