# Supplementary material for: Effect of Targeted Cytokine Inhibition on Progression of Post-Traumatic Osteoarthritis Following Intra-Articular Fracture
Source: Int J Mol Sci. 2023 Sep 2;24(17):13606. doi: 10.3390/ijms241713606 (PMC10487447; doi:10.3390/ijms241713606)
Supplement: Supplementary file 1 [file ijms-24-13606-s001.zip › ijms-2554364-supplementary.pdf]

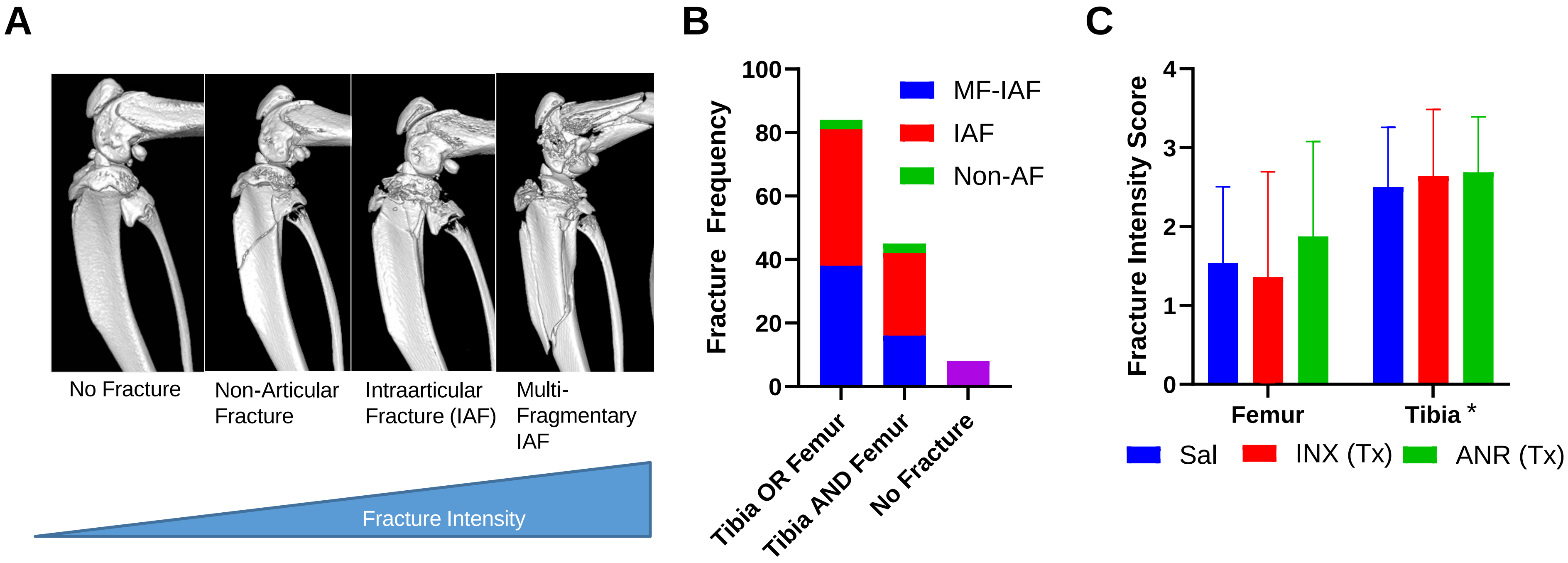

Supplemental Figure 1: Effect of Impact-Induced Trauma on IAF Generation and Fracture Intensity. (A) Representative images of the types of fractures induced by blunt-force-trauma impacts at 5J of energy. Fracture intensity increasing from left to right as defined by AOOTA compendium. (B) Aggregated data of fracture frequency from IAF or Multi-fragmentary (MF)-IAF induced in one limb bone (Tibia or Femur), both limb bones (Tibia and Femur) or non-articular fractures (non-AF, NF). (C) Fracture intensity scores by treatment group defined by AOOTA numerical classification. Values are mean ± SD of n=6 animals per group. * indicates $P<0.05$ for location (Tibia)
